# The lung cancer breath signature: a comparative analysis of exhaled breath and air sampled from inside the lungs

**DOI:** 10.1038/srep16491

**Published:** 2015-11-12

**Authors:** Rosamaria Capuano, Marco Santonico, Giorgio Pennazza, Silvia Ghezzi, Eugenio Martinelli, Claudio Roscioni, Gabriele Lucantoni, Giovanni Galluccio, Roberto Paolesse, Corrado Di Natale, Arnaldo D’Amico

**Affiliations:** 1 Department of Electronic Engineering, University of Rome Tor Vergata, Via del Politecnico 1, 00133 Rome, Italy; 2 Center for Integrated Research – CIR, Unit of Electronics for Sensor Systems, “Università Campus Bio-Medico di Roma”, via Álvaro del Portillo 21, 00128 Rome, Italy; 3 S. Camillo - C. Forlanini Hospital, Circ.ne Gianicolense 87, 00152 Rome, Italy; 4 Department of Chemical Science and Technology, University of Rome Tor Vergata, Via della Ricerca Scientifica, 00133 Rome, Italy

## Abstract

Results collected in more than 20 years of studies suggest a relationship between the volatile organic compounds exhaled in breath and lung cancer. However, the origin of these compounds is still not completely elucidated. In spite of the simplistic vision that cancerous tissues in lungs directly emit the volatile metabolites into the airways, some papers point out that metabolites are collected by the blood and then exchanged at the air-blood interface in the lung. To shed light on this subject we performed an experiment collecting both the breath and the air inside both the lungs with a modified bronchoscopic probe. The samples were measured with a gas chromatography-mass spectrometer (GC-MS) and an electronic nose. We found that the diagnostic capability of the electronic nose does not depend on the presence of cancer in the sampled lung, reaching in both cases an above 90% correct classification rate between cancer and non-cancer samples. On the other hand, multivariate analysis of GC-MS achieved a correct classification rate between the two lungs of only 76%. GC-MS analysis of breath and air sampled from the lungs demonstrates a substantial preservation of the VOCs pattern from inside the lung to the exhaled breath.

The volatile organic compounds (VOCs) exhaled by humans are supposed to be a valuable source of information about the condition of the body. A large variety of VOCs are emitted by the different body compartments: a recent review provides a list of 1840 VOCs pertinent to a healthy human body, a considerable part of which is found in the breath^1^.

Respiration brings air into the lungs with the main purpose of introducing oxygen into the body. Exhaled breath is the re-emission of the inhaled air after the subtraction of the oxygen necessary to life. With respect to inhaled air, exhaled breath is depleted of oxygen and enriched, other than CO_2_, by a number of compounds captured at the tissue-air interface all along the respiratory tract. Most of these compounds are metabolic products, so the exhaled breath may contain the footprints of cellular activities. The subsequent analysis of the breath can reveal those pathologies that alter the metabolism. Capturing information contained in breath is attracting several researchers and breath analysis is emerging as one of the most promising fields of interest for gas sensor technology^2^.

Lung cancer, in particular, attracted a large interest for its obvious implications with the breath^3^ and all these studies support the hypothesis that lung cancer alters the breath composition^4^,^5^,^6^,^7^.

Nevertheless, the results about the identification of the relevant volatile compounds are rather sparse and scarcely comparable, probably because of a lack of standardization of breath sampling and analysis. For instance, in a survey of 10 papers on the subject, 170 different detected VOCs were counted in total but only 17 of them appeared in at least two different experiments^8^.

All these studies shown that, while it is not possible to identify specific markers for lung cancer, the disease alters the concentration of a manifold of compounds, modifying the overall chemical composition of the breath. This is a typical situation where arrays of partially selective gas sensor arrays (also known as electronic noses) can be fruitfully applied^9^.

Di Natale *et al.* obtained a first result about the possibility to diagnose lung cancer with a gas sensor array composed of porphyrins coated Quartz Microbalances (QMB)^10^. This result was corroborated by a successive investigation aimed at extending studied cases, introducing comorbidities such as the Chronic Obstructive Pulmonary Disease (COPD)^11^.

The results obtained by the porphyrins based QMB array were confirmed and expanded in a number of different experiments using various sensor technologies. The case of sensors based on the conductivity changes of a layer of gold nanoparticles (GNP) coated with various functional organic molecules, such as alkanethiols, is particularly interesting^7^. The same sensor array was also demonstrated to be able to identify lung cancer cell lines from normal lung cell cultures^12^. The potentialities of the organically capped GNPs have been further demonstrated, identifying both first-stages and post tumor resection cases^13^.

Other positive results in the identification of lung cancer were achieved with arrays of polymers embedded carbon black resistors^14^,^15^ and with arrays of colorimetric sensors based on porphyrins and acid-base indicators^16^,^17^.

In spite of the growing number of papers showing the distinct composition of the breath of lung cancer affected individuals, the origin of the involved VOCs is still debated. The most plausible causes for breath modification include oxidative stress, gene mutations and the Warburg effect^18^.

In this paper the origin of VOCs in the case of lung cancer is investigated with a GC-MS and an electronic nose by measuring the air collected from inside both the lungs with a modified bronchoscopic probe. The two samples are also compared with the breath collected from the same patients. To the best of our knowledge, the collection of air from the lung carrying the cancer (ipsilateral lung) and the non-affected lung (contralateral lung) was previously reported by two groups, which exploited the one-lung ventilation during lung-surgery^19^,^20^. With respect to these studies, the investigation reported here is extended to non lung-cancer cases, thus it allows one to study the diagnostic properties of breath collected from affected and non affected lungs.

The analysis of GC-MS data indicates a slight difference between the lung hosting the cancer and the lung not affected by cancer, while the gas sensor array does not distinguish between the lungs. On the other hand, the sensor array obtains an excellent identification between cancer and non-cancer situations, by measuring the exhaled breath and the air sampled in the lungs, independently on the location of the cancer. Preliminary results from this experiment, but limited only to a subset of electronic nose data, were previously published^21^.

The electronic nose was an array of metalloporphyrins coated QMB of the same kind previously used for lung cancer detection^10^,^11^. The role of porphyrins as sensitive materials for cancer detection is intriguing, considering that oxidative stress is one of the major causes of the production of cancer related VOCs. Oxidative stress is the result of the increased activity of the cytochrome P450 enzyme, whose prosthetic group is an iron porphyrin (the heme)^22^. Therefore, the molecule used by the organism to produce the volatile compounds may also be used to detect them.

## Results

The experiment involved 30 subjects. Lung cancer was diagnoses for 20 of them while the others, although negative to cancer, were affected by other lung ailments. The demographics of the involved subjects are shown in Table 1.

For each subject air samples were collected from different portions of the respiratory tract. In-situ sampling with the modified bronchoscope provided samples from ipsilateral and contralateral lungs. Furthermore, the breath of each individual was sampled, before the bronchoscopic exam, separating the upper portion of the breath (the so-called dead space) from the deepest part that is likely to be related to the alveolar breath. The separation of the breath does not ensure a complete removal of the effects of the dead space. In particular, the addition to the breath of molecules produced in the upper airways is not eliminated. It follows that the second portion of the breath, rather than alveolar breath, is here more properly called mixed-expired breath.

The samples were differently distributed between the GC-MS and the electronic nose. All the samples, except the dead space, were measured with the electronic nose. The GC-MS was used to measure all the four kinds of samples, but only for the subset of cancer affected individuals.

### Electronic nose

The electronic nose was used to measure the mixed-expired breath and the air sampled from inside the lungs. In all cases, the electronic nose signals were used to classify cancer with respect to non cancer cases.

The Partial Least Squares Discriminant Analysis (PLS-DA) applied to the electronic nose data related to mixed-expired breath resulted in a 93% correct classification rate with a three latent variables model. The number of latent variables was determined with a leave-one-out procedure aimed at minimizing the prediction error. Due to the esiguity of the data set, the PLS-DA models here discussed have not been validated by an independent data-set. Nevertheless, it has been demonstrated that cross-validated PLS-DA is equivalent to a multivariate ANOVA statistical test^23^ with additional benefit of scores plot where the mutual relationship among the data can be visually appreciated.

**Figure 1:**
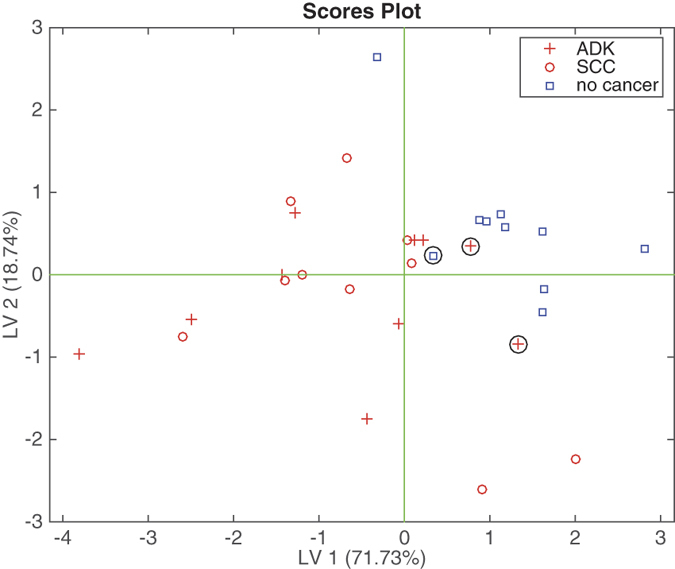
Scores plot of the first two latent variable of the PLS-DA model aimed at classifying cancer and non-cancer from the electronic nose data related to the mixed-expired breath. ADK and SCC cases are also marked. Circles mark the wrongly classified data.

The plot of the first two latent variables, shown in Fig. 1, provides a visualization of the electronic nose data clustering. This result is rather coherent with the previous findings with electronic noses using the same sensor technology^10^,^11^. In particular, as found in ref. 11, where the same breath sampling procedure was adopted, the tendency of cancer data to be more scattered than the controls is confirmed.

It is important to highlight that in this case the control group of non-cancer individuals was actually affected by various pathologies, whose symptoms required an endoscopic exam of the lung. The class of cancer affected individuals contains a mix of adenocarcinomas (ADK) and squamous cancer cells (SCC). The classifier was trained to distinguish between cancer and non cancer, so the separation between ADK and SCC is not pursued and the scores plot shows a clear overlap between the two kinds of cancer.

The application of PLS-DA only to the cancer data did not obtain more than 56% of correct classification between ADK and SCC. The lack of separation between ADK and SCC indicate that the difference of VOCs concentration that should distinguish between ADK and SCC is smaller than the resolution of the sensors. On the other hand, to the best of our knowledge different VOCs patterns between ADK and SCC have been measured in histological samples^24^.

The main objective of this paper is to study the relationship between the VOCs sampled in the lung and the cancer itself. PLS-DA models have been calculated with the data related to the samples collected in the lungs. In both cases, two latent variables minimized the prediction error calculated with the leave-one-out cross-validation.

**Figure 2:**
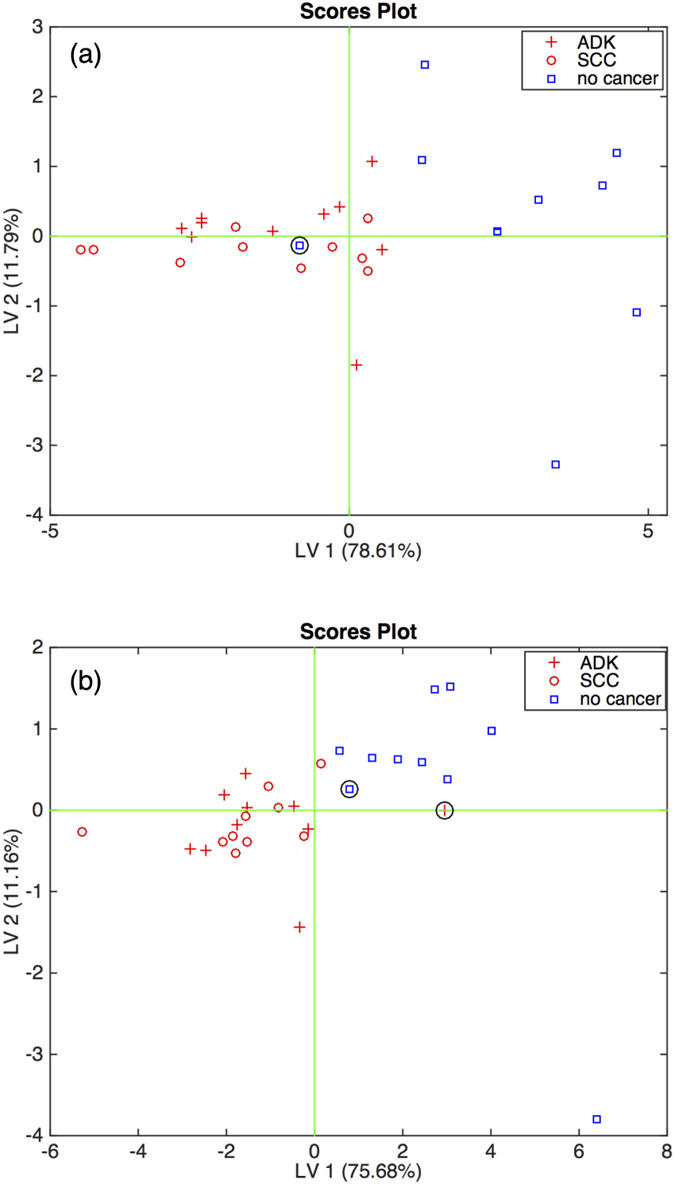
Scores plot of the first two latent variable of the PLS-DA model of classifying cancer and non-cancer from the electronic nose related to the air sampled from inside the affected lung (Fig. 2a) and not-affected lung (Fig. 2b). ADK and SCC cases are also indicated. Circles mark the wrongly classified data.

Surprisingly, the classification results are independent of the lung from where the samples have been collected. The percentage of correct classifications achieved with the mixed-expired breath is approximately the same as those obtained with the samples collected from inside the lungs. The rate of correct classifications is slightly superior with the air collected from the contralateral lung. The performance of the classifiers is 93% of correct classification for the affected lungs and 96% for the non-affected lungs. The score plots of the first two latent variables with the samples collected from the ipsilateral and contralateral lungs are shown in Fig. 2a,b respectively. It is remarkable that, with the same porphyrins coated QMB array, the mixed-expired breath and the samples collected inside the lungs achieve the same performance in the identification of lung cancer.

A comparison of the sensors patterns of misclassified samples with the mean pattern of each class (see Suppl. Fig. 1, Suppl. Fig. 2, and Suppl. Fig. 3) shows that the signals of some of the sensors are compatible with the class at which the sample belong, while other sensors of the array provide signals different from the average expected for the class. Although a thorough investigation of the relationship betwen individual sensor signals and cancer is beyond the scope of this paper, however the sensors pattern of the misclassified samples suggest that the misclassifications are due to the alteration of some compound to which some of the sensors are more sensitive and then a careful design of the sensor array might likely eliminate the classification errors.

**Figure 3:**
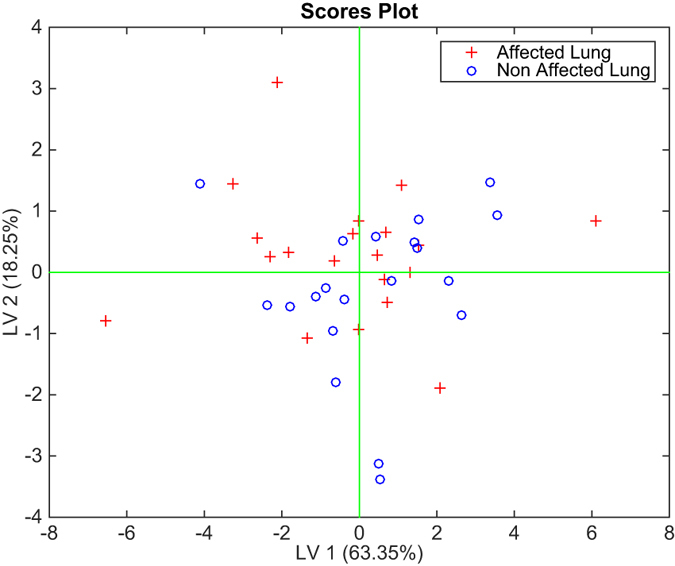
Scores plot of the first two latent variable of the PLS-DA model classifying cancer affected lungs and non cancer-affected lungs from the electronic nose related to the air sampled from inside the lungs.

Any attempt to classify the data in order to separate the affected from the non-affected lung in the case of cancer is rather unsuccessful. PLS-DA shows a rate of correct classifications of around 60% with a 2 latent variables model. Figure 3 shows the plot of the first two latent variables, where the mix between affected and non-affected lung is clearly appreciable.

### Gas chromatography

GC-MS analysis was applied to samples collected from inside the lungs, the mixed-expired breath and the dead space of cancer affected individuals. Compounds released also by the sampling material (in particular the bronchoscope) have not been considered (a list of background compounds is provided in Suppl. Table 1 of supplementary information).

Seventeen compounds, listed in Table 2, related to lung cancer, were identified by comparison with the instrument mass spectra libraries. For each compound the reliability of the identification is given as a percentage in Table 2. These compounds are plausible components of breath and some of them have been found as characteristic VOCs in lung cancer. For instance, ethanol, toluene, 2-butanone, and 2,2-dimethyl-hexanal, were found in anomalous concentration in the breath of lung cancer affected persons^7^,^19^, while cyclohexanone, undecane and toluene were found in lung cancer pleural effusions^25^. Thymol is likely derived from food or drugs.

It is important to emphasize that the identification of compounds in different experiments is rather complicated, because of the different methodologies for VOC capture (e.g. SPME fiber composition, exposure time and temperature) and the different setup of the GC-MS instrument.

**Figure 4:**
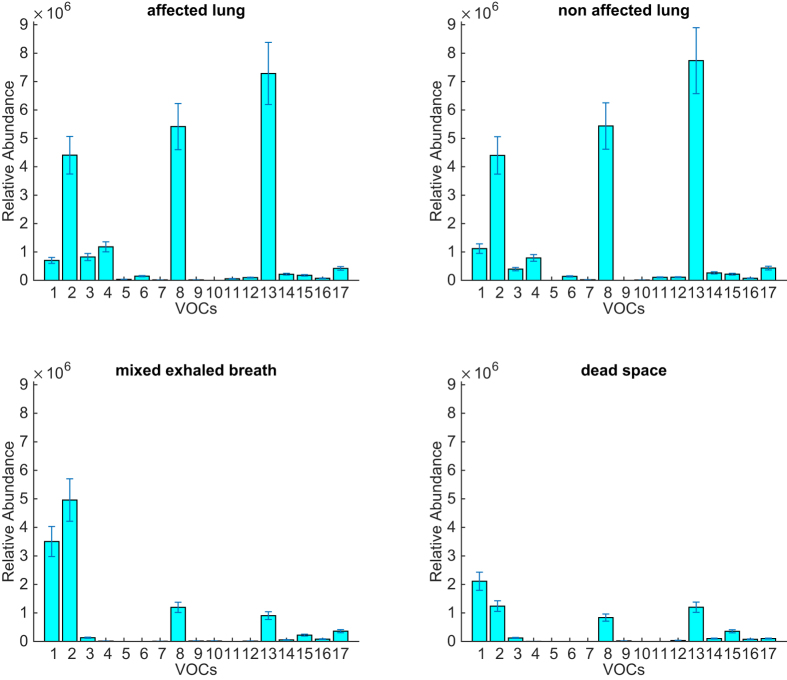
Average abundance of the VOCs listed in Table 2 and found in the four kinds of measured samples.

Figure 4 shows the average abundance of the compounds in the four compartments: affected lung, non-affected lung, mixed-expired breath, and dead space.

The plot shows a substantial similarity between the profiles of the two *in-situ* collected samples. Many of the compounds present in the lungs are also found in the mixed-expired breath and the dead space. A general decrease of abundance is observed for all the compounds, except ethanol, whose abundance is greater in the breath than in the lung. This indicates a non negligible production of this compound in the upper respiratory tract, on the other hand, ethanol is suggested as a probable marker of head and neck tumors^26^,^27^.

These results corroborate the electronic nose results and indicate that the breath collection method preserves some of the features of the VOCs pattern. However, it has to be noted that the relative abundance of some compounds (in particular compounds 8 and 12 in Table 2) is attenuated in the mixed-expired breath with respect to the lungs. So the VOCs pattern originated in the lungs may undergo a distortion along the airways. Nevetheless, these changes do not affect the electronic nose’s capability to distinguish cancer with respect to non-cancer.

It is interesting to investigate if the GC-MS is able to capture any differences between the two lungs of the same patient. In Table 2 the p-value from the Kruskal-Wallis rank test applied to each VOC is reported. No individual VOCs can be considered significantly different in the two lungs.

**Figure 5:**
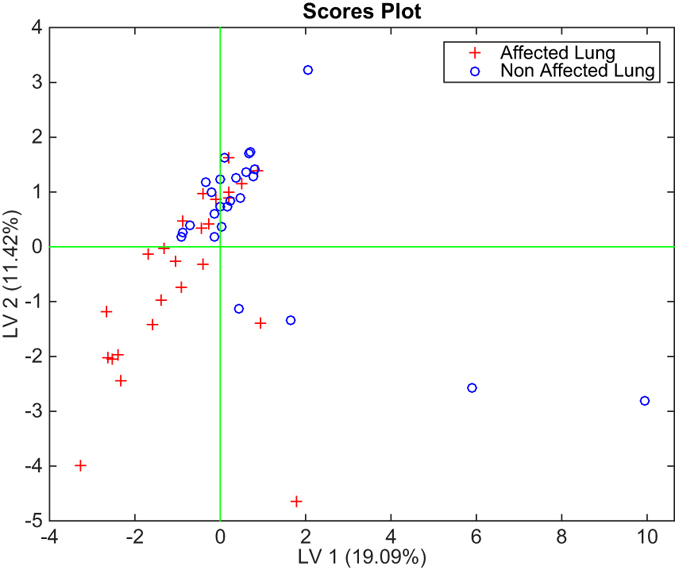
Scores plot of the first two latent variable of the PLS-DA model of classifying cancer affected lungs and non cancer-affected lungs from the abundances of the 20 compounds listed in Table 2 in the samples of air collected from inside the lungs.

However, even if the single VOCs do not discriminate the two lungs, the classification could be achieved by a multivariate classifier. To this purpose a PLS-DA classifier, based on the pattern formed by the 20 identified compounds, was calculated. The classifier achieveed a 76% correct classification rate between the two lungs. The prediction error evaluated with the leave-one-out cross-validation procedure was minimized by 4 latent variables. Figure 5 shows the plot of the first two latent variables.

These data indicate that a certain difference between the lungs exists and thus a minor portion of VOCs is directly exhaled from the cells in the breath. On the other hand, this quantity is too tiny to give rise to a complete discrimination by GC-MS and to be captured by the sensors of the electronic nose.

These results are in partial agreement with the results of Wang *et al.*^20^, where VOCs were sampled from both lungs exploiting the one-lung ventilation during the surgical resection of tumor. PLS-DA in that experiment obtained a complete discrimination between lungs before tumor resection. It is interesting that Wang *et al.*^20^ used the same SPME and GC-MS equipment used in this paper. However, in this paper the samples were collected during the bronchoscopic exam, with the patients in a completely different situation.

## Discussion

The lung cancer identification properties of the electronic nose in large part do not depend on the lung from which the sample is collected. It is important to remark that this result could be due to the lack of sensitivity of the sensors used in this experiment to some key compounds characteristic of the affected lung. It is however worthy of note that the above conclusions hold for the specific electronic nose used in this investigation. On the other hand, GC-MS analysis shows that a small difference between the two lungs can actually exist, even if only 76% of correct identification between the two classes of lungs has been achieved.

Here we also investigated how VOCs released in the lung propagate along the airways. A comparison between breath (dead space and mixed expired breath) and lungs show a substantial preservation of the VOCs, whose relative abundance is more diluted in the breath, with the exception of ethanol that is probably produced in the upper airways. Interestingly, the dilution factor is not the same for all the compounds. This leads to a distortion of the pattern that could be important for electronic nose sensors, whose signal depends on the VOCs pattern. In this paper, cancer and non-cancer samples have been separated with the same efficiency measuring the air from lungs and breath. However, we cannot exclude that these differences might be important when the classification for other pathologies is attempted. A deeper investigation into the relationship between the dilution factor and the molecular properties of the VOC is necessary for an adequate understanding of how the VOCs propagate and how to optimize the measurement method.

It is important to consider that the sampling method involves the collection of breath in bags kept at room temperature. In experiments discussed here tedlar bags were used. Tedlar is a commonly accepted material for breath collection, with superior properties in terms of background emission and VOCs stability^28^. However, the drop of temperature between the air inside the body and the external temperature should induce condensation phenomena that tend to reduce the concentration of the less volatile compounds. Furthermore, the inertness of the bag material is not absolute so we cannot exclude that some compound might stick at the surface. The measurement protocol has been standardized in order to maintain these interferences constant. The measurement room was properly climatized and the residence time of the sampled air in the bag has been rendered homogeneous.

The results of this paper support the hypothesis that the volatile products of metabolism are mainly released through body fluids and in particular blood, from where they reach the different body tissues^29^. The successive release of the metabolism products depends on the partition coefficients at the interface between the blood and the tissues. It is for this reason that the simultaneous measurement of VOCs from different body compartments is suggested to measure the global volatile metabolic products^30^.

In the lungs at the blood-air interface the volatile metabolic compounds may be displaced from the blood to the air and eventually released in the breath. According to this scenario, the VOCs are emitted at the blood-air interface independently of the location of the cancer. This finding is coherent with the previous results about the identification in the breath of breast cancer with GC-MS^31^ and of colon and prostate cancer with an array of organically capped nanoparticles^32^,^33^.

In conclusion, this study confirms the power of breath analysis in signaling lung cancer, but it also suggests that cancer related VOCs may likely be non-specific for a given kind of cancer, but they may be rather indicative of cancer diseases occurring in some non identified body compartment.

## Methods

### Study population

The experiments were carried out at the Thoracic Endoscopy Center of the San Camillo-Forlanini hospital in Rome. For the study a total of 30 subjects were enrolled: 20 affected by lung cancer and 10 affected by other lung diseases. Noteworthy, for all of them as a result of X-ray and/or X-ray computer tomography analyses a lung endoscopy was required. Prior written informed consent was provided by all participants. Consent was obtained according to the Declaration of Helsinki and all aspects of the study were approved by the research ethics committee of the San Camillo - Forlanini hospital in Rome. All the experiments were carried out in accordance with the guidelines approved by the ethics committee.

### Sample collection

Air samples were collected in the lungs by means of an endoscopic probe using the tubing access for the bronchoalveolar lavage. The main features of the sampler have been previously described^21^.

The breath was collected separating the upper and the deep portions of each single breath. The split of breath in two parts is based on the assumption that the last portion of a deep breath mainly contains the air coming from the alveoli^34^. The breath sampler was made by two sterile Tedlar (polyvinylfluoride) bags of different volume, connected to a mouthpiece. The small bag was always kept open, and access to the large bag was regulated by a three-way valve (Quintron QT00854/5-P). The first portion of breath directly fills the small bag, and, when full, the increased resistance of the air opens the valve, redirecting the expired air into the largest bag. Eventually, the large bag contains the deep breath supposed to be representative of the alveolar breath. In the present experiment a volume of 0.5 L was selected for the first bag, so collecting in the second bag (3 L) the alveolar portion of breath or, since the effect of upper airways still persists, the mixed-expired breath. Previous studies of dead space indicated a mean value of about 130 mL, but considering the large variability among subjects, a dead volume about four times larger was considered^35^. The removal of a dead space of 0.5 L was demonstrated to enhance the electronic nose identification of lung cancer^36^.

The bags were endowed with the necessary fixture for the connection to the electronic nose and to the sampler for the gas chromatography. The efficiency of the breath sampler was discussed elsewhere^12^.

### Gas chromatography

The composition of gas samples was measured with a GC-MS (Shimadzu, QP2010). The VOCs transfer was performed with a Solid Phase MicroExtraction (SPME) fiber (Supelco SPME Fiber Assembly 75 μm Carboxen-PDMS). The SPME fiber was inserted in the sampling bags and kept in place for 2 hours at room temperature.

GC-MS working conditions were as follows: the injection port temperature was set at 250°C, the oven temperature started from 40°C, and increased at the rate of 10.0°C/min up to 210°C, then at the rate of 60.0 °C/min until 300 °C were reached and held for 6 min. The column was Equity-5 capillary and the carrier was a steady flow of helium (5.2 mL/min).

Both the ion source and the interface temperature of the mass-spectrometer were fixed at 250°C. The detector voltage was 0.7 kV and the measured mass to charge ratio was between 80 and 250 m/z.

Chromatogram curves were processed to calculate the abundance of each peak, using the post-run analysis section of the GC-MS solutions software (version 2.4, Shimadzu Corporation). The identification of the compounds was done comparing the mass spectra with both NIST 127 and NIST 147 libraries. Silicon containing compounds were considered as degradation products of the SPME fibers and then discarded from the successive analysis.

### Electronic nose

The electronic nose was an ensemble of eight quartz microbalance (QMB) gas sensors. In these sensors, a slight mass change (Δm) on the quartz surface results in frequency changes (Δf) of the electrical output signal of the oscillator circuit, at which each sensor is connected. The quantities Δm and Δf are linearly related to each other in the low-perturbation regime^37^. The QMBs had a fundamental frequency of 20 MHz, corresponding to a mass resolution in the order of a few nanograms.

Each QMB was functionalized with a different layer of metalloporphyrin. Metalloporphyrins are versatile molecules that can host several interaction mechanisms, from weak and non-selective dispersion forces to the more specific coordination on the central metal ion. The balance between these forces can be controlled by the structure of the peripheral groups and the metal ion coordinated to the porphyrin ring. Then, metalloporphyrins with different sensitivities and selectivities can be obtained^38^ and assembled into sensor arrays for electronic noses^39^.

During each measurement session the sensors were continually kept under a constant flow of synthetic air and the inlet was switched to the sample for a limited amount of time, compatible with the amount of collected air. The exposure time was standardized to 3 minutes for all the cases.

The sensor response was evaluated as the frequency shift between the resonance frequency measured immediately before the exposure and at the end of the exposure. The mixed-expired breath and the air from inside each lung collected from the same individual were measured immediately after the samples collection.

### Data Analysis

The responses of the eight sensors were assembled in a vector and the whole experiment resulted in a matrix of data 30 (patients) * 8 (sensors). The data were used to patients according to the presence or not of cancer in the lung. For this aim the partial least squares discriminant analysis (PLS-DA) algorithm was used^40^. The optimal number of latent variables was found minimizing the cross-validation error in a leave-one-out cross validation procedure. The same PLS-DA algorithm was used to classify the pattern of relative abundances collected by GC-MS. In this case the sensors in the data matrix were replaced by the abundance of each identified peak.

The statistical difference between the abundances of the VOCs measured in ipsilateral and contralateral lungs was evaluated with the non-parametric Kruskal-Wallis rank sum test.

All calculations were performed in Matlab.

## Additional Information

**How to cite this article**: Capuano, R. *et al.* The lung cancer breath signature: a comparative analysis of exhaled breath and air sampled from inside the lungs. *Sci. Rep.* **5**, 16491; doi: 10.1038/srep16491 (2015).

## Supplementary Material

Supplementary Information

## Figures and Tables

**Table 1:** Demographical data the study population.

	Number	Age	Smoker (Current; Ex; Never)	Sex (M; F)	Diagnosis (ADK; SCC; OTHER respiratory diseases)
Positive	20	67 (±9)	6; 10; 4	13; 7	(15; 5; 12)
Negative	10	64 (±7)	5; 2; 3	4; 6	(0; 0; 10)

**Table 2:** List of the compounds in the GC-MS of our samples collected in the two lungs, in the mixed-expired breath and in the breath dead space.

Order	Retention Time [min]	CAS #	Compound	Percentage of identification	p-value between lungs
1	2.38	64-17-5	Ethanol	99%	0.2278
2	3.05	78-93-3	2-Butanone	98%	0.8942
3	3.86	110-02-1	Thiophene	85%	0.3214
4	5.47	123-19-3	4-Heptanone	80%	0.6961
5	5.73	105-54-4	Butanoic acid, ethyl ester	93%	0.0678
6	6.46	13831-30-6	Acetic acid, (acetyloxy)-	88%	0.6210
7	7.09	108-94-1	Cyclohexanone	97%	0.4125
8	7.75	58037-87-9	Bicyclo[3.1.0]hexane, 4-methyl-1-(1-methylethyl)-, didehydro deriv.	88%	0.4218
9	7.80	996-12-3	Hexanal, 2,2-dimethyl-	87%	0.0738
10	7.96	3842-03-3	Butane, 1,1-diethoxy-3-methyl-	94%	0.0399
11	8.19	13442-89-2	Pentane, 1-(1-ethoxyethoxy)-	86%	0.7931
12	8.74	62016-28-8	Octane, 2,2,6-trimethyl-	89%	0.9392
13	8.95	104-76-7	1-Hexanol, 2-ethyl-	98%	0.8933
14	9.63	1120-21-4	Undecane	94%	0.8313
15	11.74	89-83-8	Thymol	88%	0.2814
16	11.93	18675-24-6	1-Decanol, 2-methyl-	95%	0.9570
17	13.04	17312-54-8	Decane, 3,7-dimethyl-	91%	0.9087

For each compound the elution time, the probability of identification and the p-value from the Kruskal-Wallis rank test aimed at differentiating the compound from ipsilateral and contralateral lungs.
