# Supplementary material for: The lung cancer breath signature: a comparative analysis of exhaled breath and air sampled from inside the lungs
Source: Sci Rep. 2015 Nov 12;5:16491. doi: 10.1038/srep16491 (PMC4642313; doi:10.1038/srep16491)
Supplement: Supplementary Information [file srep16491-s1.pdf]

The lung cancer breath signature: a comparative analysis of exhaled breath and air sampled from inside the lungs

Rosamaria Capuano[1], Marco Santonico[2], Giorgio Pennazza[2], Silvia Ghezzi[1], Eugenio Martinelli[1], Claudio Roscioni[3], Gabriele Lucantoni[3], Giovanni Galluccio[3], Roberto Paolesse[4], Arnaldo D'Amico[1], Corrado Di Natale[1+*]*

1 *Department of Electronic Engineering, University of Rome Tor Vergata, Via del Politecnico 1, 00133 Rome, Italy*

2 *Center for Integrated Research – CIR, Unit of Electronics for Sensor Systems, "Università Campus Bio-Medico di Roma", via Álvaro del Portillo 21, 00128 Rome, Italy*

3 *S. Camillo - C. Forlanini Hospital, Circ.ne Gianicolense 87, 00152 Roma, Italy*

4 *Department of Chemical Science and Technology, University of Rome Tor Vergata, Via della Ricerca Scientifica, 00133 Roma, Italy*

SUPPLEMENTARY INFORMATION

Endoscopic air sampler

The outlet of the bronchoscope is connected to an inflatable tedlar suitable for exhaled breath collection, bag kept inside a rigid box. The bronchoscope is equipped with inlet tubing connecting the tip of the probe with the ambient air. At the beginning the vacuum pump is not working, thus the pressure in the box and the pressure in the lung are equal to the ambient pressure and the tedlar bag is empty. The collection of air inside the tedlar bag is due to a decrease of pressure inside the rigid box actuated by a pump actuated at 50 mL/min. The difference of pressure between the inside of the bag and the box inflates the tedlar collecting the air from the lung.

Table S1: GC-MS analysis of sampling system background compounds released by the bronchoscope and collected in the sampling bag

Retention Time (min)	Area	Compounds
6.691	116520	Acetamide, N,N-dimethyl-
7.510	389580	1,3-Propanediol, 2,2-dimethyl-
8.230	544358	Phenol
8.650	2713	Formamide, N-formyl-N-methyl-
8.821	922923	1-Hexanol, 2-ethyl-
9.425	37487	Pentane, 2,2,4-trimethyl-
9.690	290538	Eicosane, 3-methyl-
10.420	44684	Propanoic acid, 2-methyl-, propyl ester
11.178	104169	Cyclohexane, isothiocyanato-
14.100	26384	Pentanoic acid, 5-hydroxy-, 2,4-di-t-butylphenyl esters
14.553	143760	Cyclohexanecarboxylic acid, butyl ester

Mixed expired breath

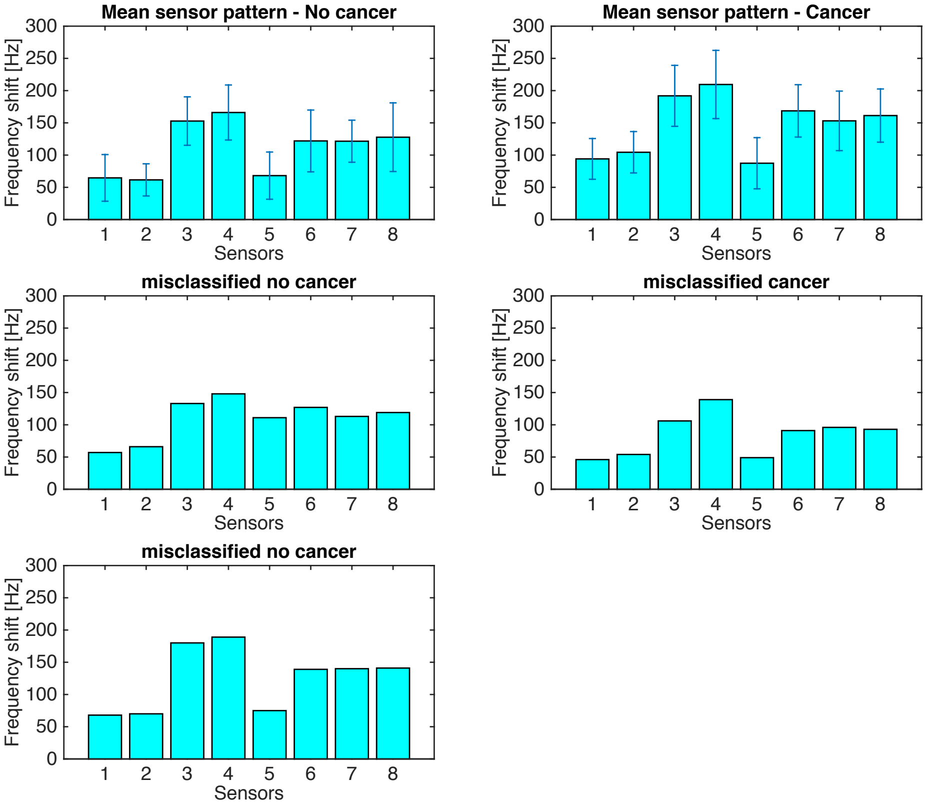

Fig S1. Mean pattern and standard deviation of no cancer and cancer related data. Patterns of the misclassified samples. The patterns of the misclassified samples are not completely different from the statistics of their class.

Ispilateral lung

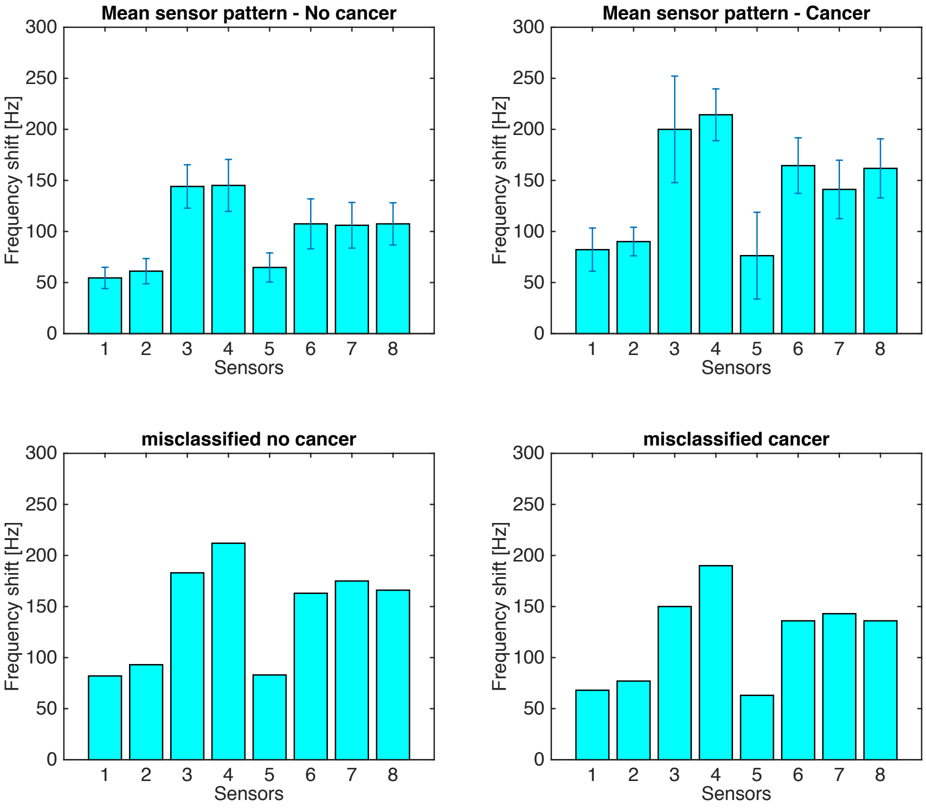

Fig. S2. Mean pattern and standard deviation of no cancer and cancer related data.
Patterns of the misclassified samples. The patterns of the misclassified samples are not completely different from the statistics of their class.

Contralateral lung

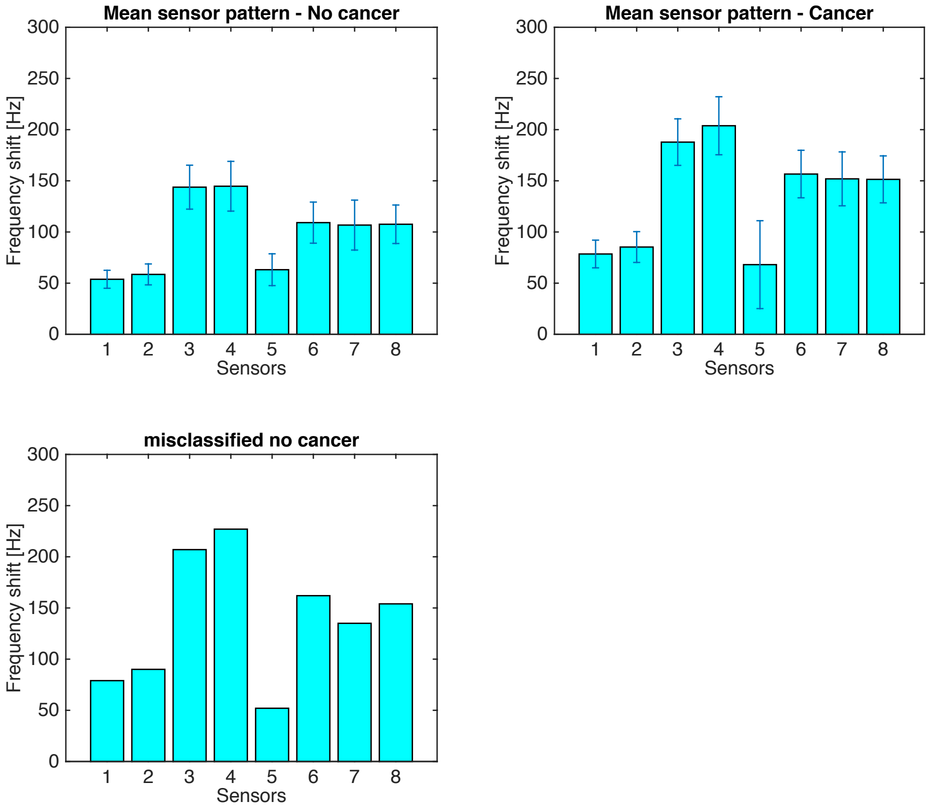

Fig. S3. Mean pattern and standard deviation of no cancer and cancer related data.

Patterns of the misclassified samples. The patterns of the misclassified samples are not completely different from the statistics of their class.
